# Higher HbA1c levels associate with lower hippocampal serotonin transporter availability in non-diabetic adults with obesity

**DOI:** 10.1038/s41598-020-78227-z

**Published:** 2020-12-07

**Authors:** Rico Grundmann, Michael Rullmann, Julia Luthardt, Franziska Zientek, Georg-Alexander Becker, Marianne Patt, Mohammed K. Hankir, Matthias Blüher, Osama Sabri, Swen Hesse

**Affiliations:** 1 Department of Nuclear Medicine, University of Leipzig, Liebigstraße 18, 04103 Leipzig, Germany; 2 Integrated Research and Treatment Center (IFB) Adiposity Diseases, University of Leipzig, Leipzig, Germany; 3 Sana Klinikum Borna, Klinik für Neurologie, Borna, Germany; 4 Department of Experimental Surgery, University Hospital Wuerzburg, Wuerzburg, Germany; 5 German Research Foundation Collaborative Research Center in Obesity Mechanisms 1052, University of Leipzig, Leipzig, Germany; 6 Department of Internal Medicine, University of Leipzig, Leipzig, Germany

**Keywords:** Preclinical research, Obesity

## Abstract

The current study aimed to investigate whether the in vivo availability of central serotonin reuptake transporters (5-HTT) is associated with plasma levels of glycosylated hemoglobin (HbA1c) in non-diabetic humans with obesity. 5-HTT availability was measured by using positron emission tomography (PET) imaging with the 5-HTT selective radiotracer [^11^C]DASB in 23 non-diabetic individuals with obesity and 14 healthy, non-obesity controls. Parametric images of binding potential BP_ND_ were generated from the PET data and analyzed together with HbA1c levels by using volume of interest analysis for brain areas relevant to appetite control. Voxel-based morphometry (VBM) of individual magnetic resonance imaging data was further performed to correlate grey matter density (GMD) maps with HbA1c. We found significant negative correlations between HbA1c levels and BP_ND_ in right and left hippocampus in obesity (r = − 0.717, p < 0.001, and r = − 0.557, p = 0.006, respectively). VBM analyses revealed that higher HbA1c levels were associated with GMD in the right para-hippocampal area. Our results indicate that chronically high blood glucose levels may evoke changes in hippocampal 5-HTT levels that are in part tied to local microstructure.

## Introduction

The pandemic spread of obesity and associated comorbidities such as type 2 diabetes mellitus (T2DM) has stimulated intense research efforts into its social, psychological as well as biological underpinnings. Although this has led to significant progress in our understanding of the central pathways that control food intake and energy metabolism, currently available obesity pharmacotherapies remain of relatively limited efficacy. Further insight into how feeding-regulatory neural circuits are targeted by central and peripheral factors, especially in human obesity, may therefore guide the development of novel treatments that cause greater weight loss.

At the molecular level, the central serotonin (5-hydroytryptamine, 5-HT) system has well-established roles in regulating energy balance. Anti-serotonergic drugs have long been known to stimulate food intake^1,2^, whereas several selective 5-HT reuptake inhibitors acting on 5-HT reuptake transporters (5-HTT), which elevate synaptic 5-HT levels, suppress food intake^3^. In addition the selective 5-HT_2C_ receptor agonist lorcaserin is an approved obesity pharmacotherapy, which also has the potential to prevent progression from prediabetes to manifest T2DM^4^. Accordingly, lorcaserin directly targets hypothalamic and hindbrain neural circuits to suppress food intake and improve glycaemic control in rodents^5,6^. It remains less clear, however, how peripheral factors influence the central serotonergic system in the context of obesiy and T2DM pathogenesis, especially in humans.

Brain-derived neurotrophic factor (BDNF) is a neurotrophin that has an influence on energy metabolism, insulin sensitivity^7^ and glucose metabolism^8^, as well as on hippocampal microstructure by altering central 5-HT signaling^9^. Recently, we investigated the link between BDNF and the genetic regulation of central 5-HTT availability^10^. We showed a significant negative correlation between BDNF and hippocampal 5-HTT availability in homozygous L (L/L) carriers who have higher 5-HTT activity^11^. BDNF might thus act as an integrative key signal at the interface of glucose metabolism and 5-HT dependent microstructure changes in hippocampus, which in turn could affect memory formation. It is therefore of interest whether there is a connection between BDNF and glycosylated hemoglobin levels (HbA1c), the variant of hemoglobin, chemically bound to glucose as a stable marker of blood glucose levels over a time period of approximately three months.

To the best of our knowledge, only one previous clinical study investigated central 5-HTT availability in lean and individuals with obesity and with or without insulin resistance, using single-photon emission computed tomography (SPECT) with the monoamine transporter-binding radiotracer [^123^I]FP-CIT. This study showed that radiotracer binding to 5-HTT in the diencephalon was lower in insulin-resistant individuals independently of body weight whereas hypothalamic 5-HTT binding was lower in individuals with obesity independently of insulin sensitivity^12^. However, the association between plasma HbA1c and central 5-HTT availability has not been explored so far in-vivo with positron emission tomography (PET) in humans.

The main aim of the current study was therefore to investigate whether there is an association between central 5-HTT availability and plasma HbA1c levels in individuals with obesity but without manifest T2DM and in healthy controls without obesity. We hypothesized that 5-HTT availability is negatively associated with plasma HbA1c in brain areas that are relevant for energy homeostasis and food intake control, e.g. the hypothalamus and the prefrontal cortex, as well as for memory formation, i.e. the hippocampus. By applying PET and the radiolabeled 3-amino-4-(2-dimethylaminomethylphenylsulfanyl)-benzonitrile ([^11^C]DASB), which binds to presynaptic 5-HTT with high selectivity, we calculated non-displaceable binding potential BP_ND_ as a marker for 5-HTT availability. We additionally performed voxel-based morphometry (VBM) analyses of magnetic resonance imaging (MRI) data to assess microstructural brain changes based on grey matter density (GMD) maps in association with plasma HbA1c levels^13^. Finally, we performed correlative analyses of HbA1c levels and serum BDNF levels in 5-HTT genotype stratified subgroups, i.e., L- and S-allele carriers^10^.

## Materials and methods

### Study participants

The study cohort represents a subset of previously published data^14^ consisting individuals with obesity and non-obesity controls for whom HbA1c assessments were available. We further excluded one individual with obesity due to prediabetic metabolism (HbA1c > 5.9%). Other exclusion criteria comprised current or past neurological or psychiatric illness, positive family history for psychiatric illnesses, former psychotherapy, resistant hypertension, type 1 and type 2 diabetes mellitus, the use of centrally-acting drugs, participation in weight loss programs during the last 6 months, past or present history of alcohol misuse and/or illicit drug abuse, as well as contraindications for MRI (e.g., implanted ferromagnetic devices, claustrophobia), pregnancy, or breast feeding^14^. Informed written consent was obtained prior to study inclusion as well as detailed medical history and clinical examinations.

PET imaging data and blood samples in 23 individuals with obesity with a body mass index (BMI) of 41.7 ± 4.9 kg/m^2^ (6 men; mean age: 36.1 ± 10.2 years; range 21–56 years) and 14 healthy, non-obesity controls (BMI: 22.5 ± 2.6 kg/m^2^; 5 men; mean age, 36.1 ± 7.2 years; range 21–49 years) were analyzed.

### Imaging

All study participants received a PET scan as previously described^14^. Briefly, each study participant underwent a 90-min dynamic scan (23 frames: 4 × 15 s, 4 × 1 min, 5 × 2 min, 5 × 5 min, 5 × 10 min) using the ECAT EXACT HR + tomograph (Siemens, Erlangen, Germany, intrinsic resolution at the center: 4.3 mm, axial resolution: 5–6 mm field of view 15.5 cm, 3–4 mm full width at half maximum) in a 3-dimensional acquisition mode. [^11^C]DASB was synthesized according to the procedures reported by Wilson et al.^15^. With the start of each scan, 484.1 ± 9.9 MBq [^11^C]DASB were injected intravenously as a continuous bolus over 90 s. A 10-min transmission scan (from three ^68^ Ga/^68^Ge sources) prior to PET acquisition was used for attenuation correction and iterative reconstruction (10 iterations, 16 subsets) in transverse image series (63 slices, 128 × 128 matrix, voxel size 2.6 × 2.6 × 2.4 mm^3^) with a Hann-filter (cut-off 4.9 mm) for post-processing was applied. MRI for the exclusion of structural brain abnormalities and for imaging data co-registration was performed on all study participants. T1-weighted MP-RAGE sequences were acquired on either a Siemens Verio or TimTrio 3 T scanner (Siemens, Erlangen, Germany).

### Imaging data analysis

We co-registered a summed image of the first 13 frames of the PET data with individual 3D T1-weighted MP-RAGE MRI data, using PMOD software (Version 3.4) for re-alignment and stereo-tactical normalization according to the anterior posterior commissure line. Parametric images of BP_ND_ were generated from PET data by using a multi-linear reference tissue model with two parameters and the cerebellar cortex as a reference region. Regional analysis of BP_ND_ values was performed by atlas-based manual delineation of volumes of interest (VOI), including the dorsolateral prefrontal cortex, the orbitofrontal cortex, the insula, the ventral striatum, the thalamus, the hypothalamus, the hippocampus, the midbrain, i.e. the substantia nigra (SN) and ventral tegmental area (VTA) and the raphe nuclei.

VBM analyses of MRI data to consider micro-structural changes were performed using VBM8 (http://www.neuro.uni-jena.de/vbm/) implemented in SPM8 (Statistical Parametric Mapping, Wellcome Department of Cognitive Neurology, UK) and Matlab 7.14.0 (The MathWorks Inc., USA). In agreement with general practice, we used the term GMD to describe the normalized grey matter probability values.

### Blood plasma analysis

We collected venous blood from all study participants immediately before the start of the PET scan. Plasma was immediately collected by centrifugation and refrigerated for subsequent HbA1c (non-diabetic: ≤ 5.9%) and BDNF measurements. We excluded one individual with obesity due to prediabetic metabolism (HbA1c > 5.9%) resulting in samples from 23 participants with obesity and 14 non-obesity controls for final analysis.

### Statistical analysis

The data were analyzed using SPSS 22 software (SPSS Inc., Chicago, IL). All data are presented as mean and standard deviation. After testing our data for normal distribution using Shapiro-Wilks test (p > 0.05) Pearson product moment correlation was used to test for an association between blood parameters and 5-HTT BP_ND_ as well as volume values from our VOI measurements, respectively. Contributing to our previous study^10^, we subdivided our whole cohort (obese and non-obese) genotype depended in a homozygous L (L/L) and a homozygous plus heterozygous S genotyped (S +) group for group comparisons of serum BDNF and HbA1c levels. Unless otherwise stated, statistical significance was defined as *α* < 0.05 and two-tailed. Graphical presentations were created using GraphPad Prism 7.04 (GraphPad Software Inc., La Jolla, CA, www.graphpad.com) for Windows.

For voxel-based analysis, we used an uncorrected threshold of p < 0.001 in SPM8 while correlating GMD maps (MRI) with HbA1c levels, as well as 5-HTT BP_ND_. We applied a predefined search mask of the hippocampus and the para-hippocampal area based on previous findings for GMD-HbA1c associations^16^ as provided in Automated Anatomical Labeling atlas^17^.

### Ethics statement

The study was performed after authorization by the ethics committee of the Medical Faculty of the University of Leipzig (registered under no. 206-10-08032010). The study was registered at the European clinical trial database (EudraCT 2012-000568-32) and the German Clinical Trials Register (DRKS), and was accomplished in agreement with all provisions declared by the ICH Guideline for Good Clinical Practice (GCP), the declaration of Helsinki, and the German Federal Office for Radiation Protection (registered under no. Z5-22461-2-2011-002).

## Results

### Between-group comparison of BP_ND_ and HbA1c levels

The results of HbA1c and BP_ND_ analyses are summarized in Table 1. There was no significant group-difference of HbA1c and BP_ND_ between individuals with obesity and without obesity.

**Table 1:** Demographics and 5-HTT BP_ND_ from volume-of-interest (VOI) analysis obtained with [^11^C]DASB and PET.

	Obesity^a^	Non-Obesity^b^	p value^c^
N	23	14	
**Demographics**			
Sex (women/men)	17/6	9/5	0.71
Smoking habits (no/occasionally/yes)	13/1/1	17/2/6	0.40
Age (years)	36.1 ± 10.2	36.1 ± 7.2	0.996
BMI (kg/m^2^)	41.1 ± 4.9	22.5 ± 2.6	** < 0.0001**
HbA1c (%)	5.57 ± 0.30	5.54 ± 0.30	0.5
**VOIs**			
Dorsolateral prefrontal cortex right	0.24 ± 0.14	0.21 ± 0.08	0.52
Dorsolateral prefrontal cortex left	0.25 ± 0.14	0.22 ± 0.08	0.57
Orbitofrontal cortex right	0.45 ± 0.18	0.41 ± 0.10	0.43
Orbitofrontal cortex left	0.46 ± 0.17	0.40 ± 0.13	0.25
Insula right	0.66 ± 0.2	0.63 ± 0.16	0.7
Insula left	0.68 ± 0.23	0.64 ± 0.13	0.56
Ventral striatum	1.54 ± 0.39	1.56 ± 0.37	0.84
Ventral striatum	1.62 ± 0.47	1.65 ± 0.37	0.82
Amygdala right	1.32 ± 0.37	1.2 ± 0.34	0.34
Amygdala left	1.35 ± 0.4	1.27 ± 0.33	0.56
Raphe	3.44 ± 0.87	3.09 ± 0.67	0.2
Thalamus right	1.62 ± 0.51	1.48 ± 0.33	0.38
Thalamus left	1.53 ± 0.52	1.37 ± 0.39	0.32
Hypothalamus right	2.11 ± 0.55	1.78 ± 0.53	0.08
Hypothalamus left	2.09 ± 0.62	1.93 ± 0.52	0.45
Hippocampus right	0.58 ± 0.29	0.2 ± 0.13	0.47
Hippocampus left	0.64 ± 0.27	0.54 ± 0.2	0.22
SN/VTA right	1.58 ± 0.52	1.55 ± 0.52	0.89
SN/VTA left	1.58 ± 0.53	1.55 ± 0.5	0.86

*SN* substantia nigra, *VTA* ventral tegmental area.

Bold value indicates statistically significant group differences (p < 0.05).

^a^BMI > 35 kg/m^2^.

^b^BMI < 27 kg/m^2^.

^c^Student’s t-test except for sex and smoking habits (Fisher’s Exact test).

### Correlative analyses of BP_ND_ and HbA1c

**Figure 1:**
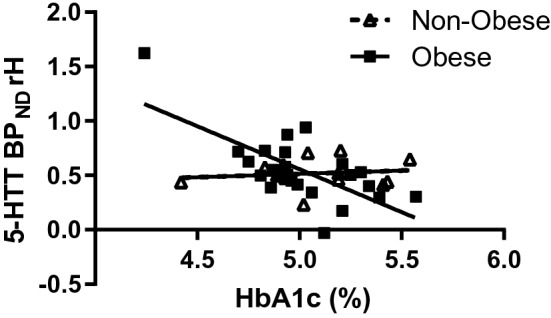
Scatterplot showing Pearson correlation between HbA1c levels and 5-HTT BP_ND_ obtained with [^11^C]DASB and PET in right hippocampus (rH) for obese (r = − 0.79, p = 0.0002) and non-obese (r = 0.06, p = 0.64). The difference of regression coefficients was significant (p = 0.002).

**Figure 2:**
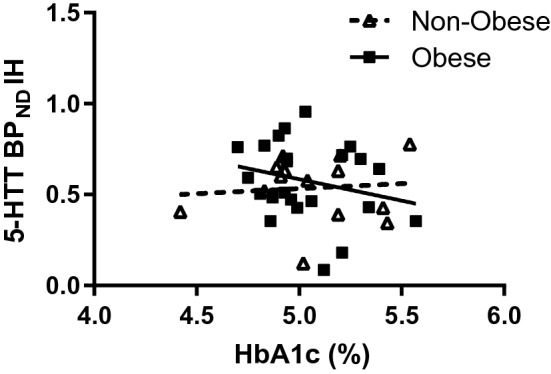
Scatterplot showing Pearson correlation between HbA1c levels and 5-HTT BP_ND_ obtained with [^11^C]DASB and PET in the left hippocampus (lH) individuals with obesity (r = − 0.57, p = 0.006) and non-obesity (r = 0.056, p = 0.76). The difference of regression coefficients was statistically significant (p = 0.03).

We found highly significant negative correlations between HbA1c levels and BP_ND_ in the right (rH, Fig. 1) and left hippocampus (lH, Fig. 2) in participants with obesity (rH: r = − 0.717, p < 0.001; lH: r = − 0.557, p = 0.006), but not in the group of healthy controls without obesity (rH: r = 0.138, p = 0.64; lH: r = 0.091, p = 0.76). The differences of the regression coefficients were significant (rH: p = 0.001; lH: p = 0.03). Partial correlations with “age” and “sex” as nuisance variable confirm the significant correlation results (“age”: rH: r = − 0.704, p < 0.001; lH: r = − 0.516, p = 0.014; “sex”: rH: r = − 0.754, p < 0.001; lH: r = − 0.561, p = 0.007). We did not find significant correlations between 5-HTT BP_ND_ and HbA1c levels in any of the other investigated VOIs.

### Voxel-based morphometry

**Figure 3:**
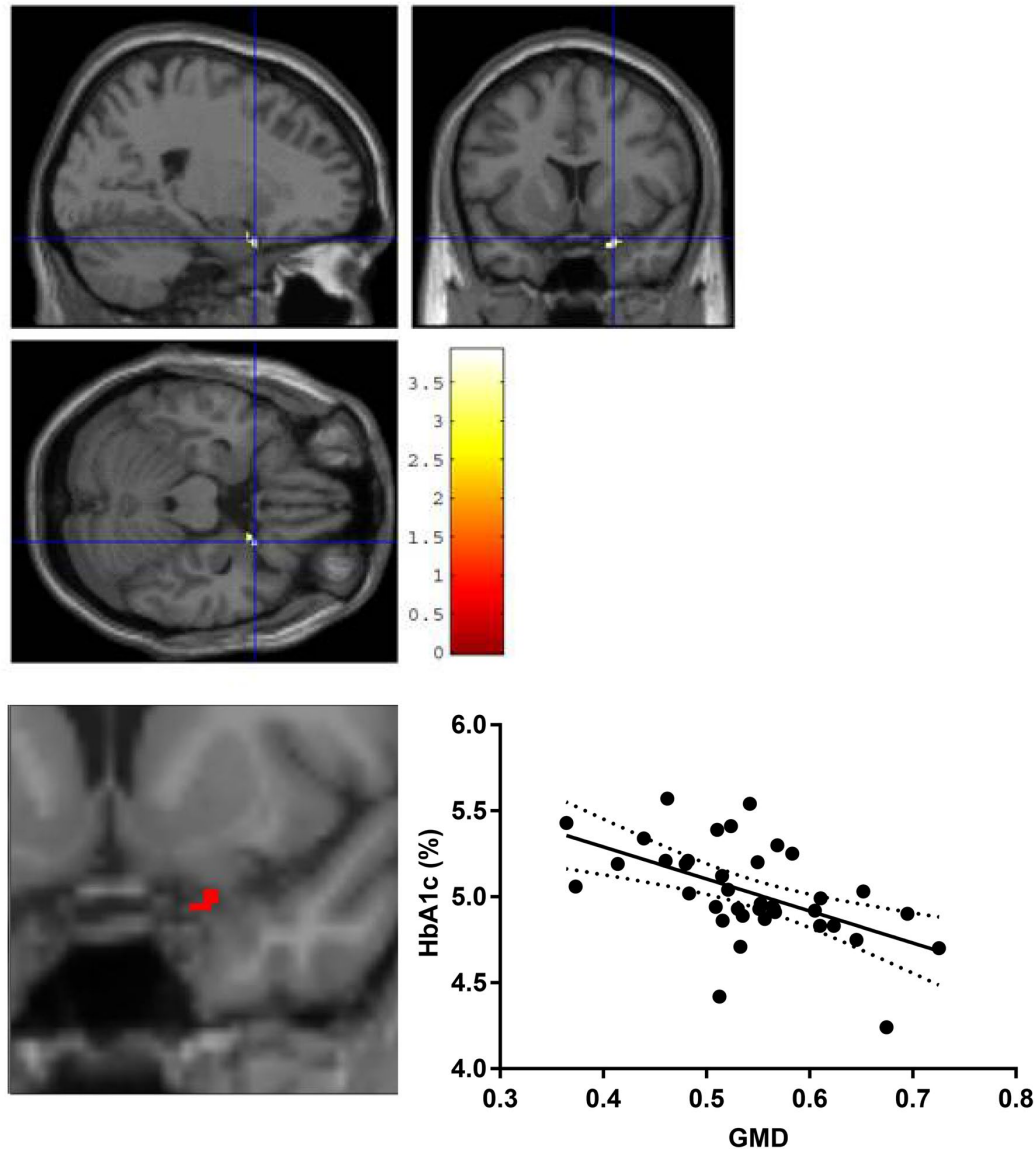
Voxel based morphometry analyses. Correlation between GMD and HbA1c, which showed clusters in parahippocampal region in the right hemisphere (18 voxels, MNI: x = 22.5, y = 11, z = − 22.5 mm, T > 3.91, p < 0.001).

Higher HbA1c levels were associated with GMD in the right para-hippocampal area (Fig. 3; MNI: x = 22.5, y = 11, z = − 22.5 mm; 18 voxels; T > 3.91, p < 0.001 uncorrected). VBM correlative analysis separated into obesity-versus-non-obesity group indicated no correlation in non-obesity, while obesity cohort showed a similar but smaller cluster of a correlation between GMD and HbA1c levels in the right hippocampus (x = 20, y = 11, z = − 23 mm; 5 voxels). There was no significant correlation between 5-HTT BP_ND_ and GMD in hippocampus or the para-hippocampal area.

### Correlative analyses of HbA1c and BDNF

**Figure 4:**
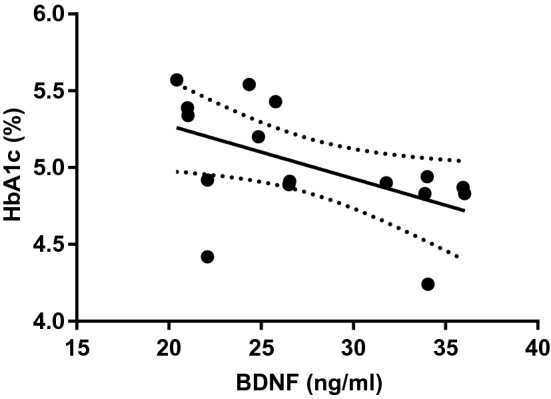
Scatterplot showing Pearson correlation between serum BDNF and HbA1c levels in S-carriers (r = − 0.531, p = 0.034).

Pearson correlation did not reveal a general relationship between serum BDNF and HbA1c levels in the whole cohort. Genotype stratified group analyses showed a significant negative correlation in the S + group (r = − 0.53; p = 0.034; Fig. 4).

## Discussion

To our knowledge, this is the first study investigating the association between the central in-vivo 5-HTT availability using ^11^[C]DASB and PET with plasma HbA1c in human obesity. We observed a significant age- and sex-independent negative effect of higher HbA1c levels on 5-HTT availability in the hippocampus of non-diabetic individuals with obesity but not in controls without obesity. VBM revealed HbA1c-dependent microstructural changes in the right para-hippocampal area. These findings contribute to the aforementioned assumption of chronically high glucose levels evoking hippocampal (micro-) structural alterations. We found a small cluster a correlation between GMD and HbA1c in the right hippocampus of our individuals with obesity but no correlation in the non-obesity group. The lack of further differences between the obesity and the nonobesity group in the voxel-based correlation is primary explained as a consequence of a small sample size. Nonetheless, our data point to a hippocampal vulnerability for increased HbA1c reflected by GMD changes and altered 5-HTT signaling in obesity that occur in the absence of, or possibly prior to, manifest T2DM.

Contrary to our initial hypothesis, we did not find correlations between HbA1c and 5-HTT availability in other brain areas that are implicated in energy homeostasis and the control of food intake, e.g. the hypothalamus and the prefrontal cortex. Altogether, differences in HbA1c levels only have an effect on hippocampal 5-HTT availability in individuals with obesity.

In can therefore be speculated whether weight gain and obesity are linked with a hippocampal-dependent process of energy dysregulation and decreased hippocampal performance. Specifically, obesity and consumption of high-fat diet can lead to impaired hippocampal cognitive functions, i.e. impaired learning about the relationship between food cues and rewarding signals, which in turn can result in attenuated cognitive control of food intake, displaying a vicious cycle of weight gain and cognitive decline^18^. In line with this, medial temporal lobe damage in humans, which involves hippocampal damage, leads to difficulties in utilizing certain information presented by their internal cues^19^, e.g. interoceptive stimuli corresponding to hunger and satiety^20^. In addition, studies reveal that inhibition of food intake to an extent depends on activation of neural pathways between the hippocampus and the lateral hypothalamus^21^. We propose that HbA1c-dependent alterations in hippocampal 5-HTT signaling in obesity is an essential part of these processes. If this is a mechanism linked to alterations in memory consolidation needs to be further tested.

Corroborating our previous findings, we showed that BDNF acts in a 5-HTT genotype-dependent manner. Here, 5-HTT genotyping analysis revealed a significant negative correlation between HbA1c and BDNF in S + subgroup. Accordingly, exercise-induced decrease in some diabetic risk factors, such as fasting glucose levels, are associated with elevated BDNF levels in adults with obesity^22^. Moreover, carriers of the functional BDNFVal66Met allele who have raised HbA1c levels showed impaired episodic memory and thus, BDNFVal66Met and elevated HbA1c might act synergistically in cognitive aging^23^. Correspondingly, an involvement of BDNF has been identified in the pathogenesis not only of T2DM, but also of dementia and depression^24^. Thus, genotype-defined modifications of central hippocampal 5-HT signaling might play a role in these frameworks of allostasis and allostatic overload^25^. However, the exact function of serotonergic actions and hippocampal formation in human obesity has yet to be clarified and tested prospectively in enlarged study cohorts with 1:1 case–control design, which could be not applied in our current study (as a major limitation). We also did not obtain fasting blood glucose, which may have an influence on study results, either. Further studies are necessary to determine the link between glucose metabolism, peripheral hormones (such as GLP-1, leptin and ghrelin), BDNF and hippocampal 5-HTT signaling and its biological relevance with regard to preventive and therapeutic consequences in individuals with obesity.

## Data Availability

All study-associated data and material is stored at the Department of Nuclear Medicine, University of Leipzig, and access is available by request.

## Abbreviations

[^11^C]DASB: 3-Amino-4-(2-dimethylaminomethylphenylsulfanyl)-benzonitrile
5-HT: 5-Hydroxytryptamine = Serotonin
5-HTT: Serotonin transporter
BDNF: Brain-derived neurotrophic factor
BMI: Body mass index
BP_ND_: Non-displaceable binding potential
GMD: Grey matter density
HbA1c: Glycosylated hemoglobin
lH: Left hippocampus
MRI: Magnetic resonance imaging
PET: Positron emission tomography
rH: Right hippocampus
SN: Substantia nigra
SPECT: Single-photon emission computed tomography
T2DM: Type 2 diabetes mellitus
VBM: Voxel-based morphometry
VOI: Volume of interest
VTA: Ventral tegmental area
